# Role of Calcium-Sensing Receptor in Mechanotransducer-Channel-Mediated Ca^2+^ Influx in Hair Cells of Zebrafish Larvae

**DOI:** 10.3389/fphys.2018.00649

**Published:** 2018-05-30

**Authors:** Li-Yih Lin, Ya-Hsin Yeh, Giun-Yi Hung, Chia-Hao Lin, Pung-Pung Hwang, Jiun-Lin Horng

**Affiliations:** ^1^Department of Life Science, National Taiwan Normal University, Taipei, Taiwan; ^2^Department of Pediatrics, Taipei Veterans General Hospital, Taipei, Taiwan; ^3^Department of Pediatrics, Faculty of Medicine, National Yang-Ming University, Taipei, Taiwan; ^4^Institute of Cellular and Organismic Biology, Academia Sinica, Taipei, Taiwan; ^5^Department of Anatomy and Cell Biology, School of Medicine, College of Medicine, Taipei Medical University, Taipei, Taiwan

**Keywords:** calcium-sensing receptor, hair cell, mechanotransducer channel, zebrafish, scanning ion-selective electrode technique

## Abstract

The calcium-sensing receptor (CaSR) is an extracellular Ca^2+^ sensor that plays a critical role in maintaining Ca^2+^ homeostasis in several organs, including the parathyroid gland and kidneys. In this study, through *in situ* hybridization, the expression of CaSR mRNA was found in the neuromasts of zebrafish larvae. Immunohistochemistry further demonstrated that the CaSR protein was present in neuromast hair cell stereocilia and basolateral membranes. Based on the expression and subcellular localization of the CaSR in hair cells, we hypothesized that the CaSR is expressed in zebrafish lateral-line hair cells to regulate mechanotransducer (MET)-channel-mediated Ca^2+^ entry. Using the scanning ion-selective electrode technique, MET-channel-mediated Ca^2+^ influx at the stereocilia of hair cells was measured in intact larvae. Ca^2+^ influx was suppressed after larvae were pretreated with a CaSR activator (R-568) or high-Ca^2+^ (HCa) medium. Gene knockdown by using morpholino oligonucleotides decreased CaSR expression in hair cells and eliminated the effects of R-568 and HCa on Ca^2+^ influx. In addition, we found that treatment with R-568 attenuated neomycin-induced hair cell death. This study is the first to demonstrate that the CaSR is involved in mechanotransduction in zebrafish hair cells.

## Introduction

The calcium-sensing receptor (CaSR), a G-protein-coupled receptor, is activated by extracellular Ca^2+^ to regulate Ca^2+^ absorption and Ca^2+^ homeostasis in several organs, including the kidneys, intestines, bone, and parathyroid gland (Brown and MacLeod, 2001; Hofer and Brown, 2003; Smajilovic and Tfelt-Hansen, 2007). In the parathyroid gland, reduced plasma Ca^2+^ levels cause a CaSR-mediated increase in parathyroid hormone (PTH) secretion (Chattopadhyay and Brown, 2006). CaSR-knockout mice displayed highly elevated PTH levels, even when plasma Ca^2+^ levels were considerably elevated, demonstrating the direct effect of the CaSR on PTH secretion (Ho et al., 1995). In the kidneys, CaSR activation inhibits both transcellular and paracellular Ca^2+^ transport in the thick ascending limb (Motoyama and Friedman, 2002). Loss-of-function mutations of the CaSR lead to decreased urinary Ca^2+^ excretion in humans and mice (Brown et al., 1998; Toka et al., 2012). In sum, these findings indicate that the CaSR plays a critical role in regulating Ca^2+^ homeostasis.

The CaSR is also involved in regulating different functions in tissues that are not related to Ca^2+^ homeostasis, such as those in the brain, pancreas, blood vessels, and heart (Smajilovic and Tfelt-Hansen, 2007). In the brain, the CaSR activates several ion channels, including non-selective cation channels (Ye et al., 1996; Chattopadhyay et al., 1999a) and calcium-sensitive K^+^ channels (Chattopadhyay et al., 1999b; Ye et al., 2004). Activation of the CaSR opens intermediate-conductance calcium-sensitive K^+^ (IKCa) channels in arterial endothelial cells (Weston et al., 2005). By contrast, an *in vitro* study demonstrated that the coexpression of the CaSR with the K^+^ channel (Kir4.1 or Kir4.2) in *Xenopus* oocytes inhibits the function of the K^+^ channel (Huang et al., 2007). Altogether, these findings suggest that the CaSR can sense extracellular Ca^2+^ and modulate the function of ion channels.

Hair cells in the inner ears of mammals are specialized mechanosensory cells involved in hearing and balance. Apical hair bundles are a special morphological feature of hair cells and consist of stereocilia that contain mechanotransducer (MET) channels (Kazmierczak and Muller, 2012). Deflection of hair bundles opens the MET channel and causes Ca^2+^ and K^+^ influx, which activates signal transduction in hair cells. An electrophysiological analysis of isolated hair cells showed that the MET channel is a non-selective cation channel with high Ca^2+^ permeability (Fettiplace, 2009). After entry through the MET channel, Ca^2+^ binds to calmodulin or acts at an unknown intracellular site to drive slow and fast adaptations (Wu et al., 1999; Peng et al., 2016). Moreover, extracellular Ca^2+^ affects the open probability of the MET channel (Ricci and Fettiplace, 1998; Farris et al., 2006; Peng et al., 2016). A study demonstrated that decreasing extracellular Ca^2+^ increased the open probability of the MET channel and amplified the blocking efficacy of aminoglycoside antibiotics (Ricci, 2002). Small organic molecules such as the fluorescent styryl dye FM1-43, which has been used as a marker of hair cell viability (Gale et al., 2001; Meyers et al., 2003; Coffin et al., 2009; Ou et al., 2010), and aminoglycoside antibiotics, which can cause hair cell death (Fettiplace, 2009; Froehlicher et al., 2009), have been found to pass through MET channels.

Ca^2+^ homeostasis is critical for the survival and functioning of hair cells during the detection and transmission of acoustic information. To maintain the intracellular Ca^2+^ concentration, hair cells contain numerous Ca^2+^-buffering proteins, such as calbindin, calmodulin, and parvalbumin (Hackney et al., 2005). Hair bundles express a plasma membrane Ca^2+^ ATPase pump (PMCA) to extrude Ca^2+^, which enters through MET channels during stimulation (Dumont et al., 2001). Disruption of intracellular Ca^2+^ homeostasis or mutations of the PMCA gene impair hair cell function and cause hearing loss (Gillespie and Muller, 2009; Bortolozzi et al., 2010). Furthermore, elevated intracellular Ca^2+^ levels have been observed in chick and mouse cochlear explants following exposure to ototoxic agents (Hirose et al., 1999; Matsui et al., 2004). In a study of zebrafish, dying hair cells exhibited a transient increase in intracellular Ca^2+^ after exposure to aminoglycosides (Esterberg et al., 2013). These data suggest that alterations in intracellular Ca^2+^ homeostasis play an essential role in aminoglycoside-induced hair cell death.

Extracellular Ca^2+^ is also crucial for hair cell function (Dumont et al., 2001; Go et al., 2010). Experiments with mouse cochlear cultures showed that elevating the extracellular Ca^2+^ or Mg^2+^ concentration suppressed neomycin-provoked hair cell damage; conversely, decreasing the extracellular Ca^2+^ or Mg^2+^ concentration enhanced the damage (Richardson and Russell, 1991). In zebrafish, increases in either extracellular Ca^2+^ or Mg^2+^ have been found to protect hair cells from neomycin-induced cell death, and the lack of external Ca^2+^ in the medium has been found to led to hair cell death (Coffin et al., 2009; Lin et al., 2013). These findings demonstrate that intra- and extracellular Ca^2+^ is critical for hair cell functioning and survival. However, the mechanism by which hair cells sense environmental Ca^2+^ concentrations and maintain an appropiate internal Ca^2+^ concentration has not yet been determined.

Inner-ear hair cells of mammals are embedded in the temporal bone, whereas zebrafish hair cells are situated in lateral-line neuromasts on the embryonic skin and can be easily observed and investigated (Ghysen and Dambly-Chaudiere, 2007). Neuromasts contain a core of approximately 15 hair cells with a structure and function similar to those of inner-ear hair cells in other vertebrates, including humans (Froehlicher et al., 2009; Ou et al., 2010). Lateral-line hair cells are also sensitive to ototoxic drugs, including aminoglycosides and cisplatin (Ou et al., 2007, 2010; Froehlicher et al., 2009). Therefore, zebrafish is a valuable *in vivo* model for studying vertebrate hair cells (Froehlicher et al., 2009; Ou et al., 2010).

CaSRs have been identified in several teleost fish organs, including the gills, olfactory organ, kidneys, and corpuscles of Stannius (Flanagan et al., 2002; Loretz et al., 2009; Lin et al., 2014). A electrophysiological study of goldfish (*Carassius auratus*) revealed that the signaling activity of the olfactory nerve increased with increasing environmental Ca^2+^ levels, presumably through CaSR activation (Hubbard et al., 2002). In a study of rainbow trout, treatment with CaSR activators stimulated the secretion of stanniocalcin, a hypocalcemic hormone, and decreased Ca^2+^ uptake (Radman et al., 2002). However, the cellular distribution and function of the CaSR in neuromast hair cells have not been investigated.

The scanning ion-selective electrode technique (SIET) has been applied to study the transport of various ions (Na^+^, Cl^-^, H^+^, NH_4_^+^, and Ca^2+^) by skin ionocytes in zebrafish and medaka (*Oryzias latipes*) (Smith et al., 1999; Wu et al., 2010; Shih et al., 2012; Horng et al., 2015). In previous studies, we also applied this non-invasive technique to detect MET-channel-mediated Ca^2+^ entry at zebrafish hair cells (Lin et al., 2013, 2015). In these studies, a Ca^2+^-selective microelectrode was used to deflect hair bundles and simultaneously record Ca^2+^ flux, and the findings demonstrated that SIET is a sensitive approach for assaying MET channels *in vivo* (Lin et al., 2013, 2015).

In this study, the SIET was used to investigate CaSR function in hair cells of intact zebrafish larvae. We hypothesized that the CaSR is expressed in hair bundles of hair cells, where this protein can sense external Ca^2+^ and regulate Ca^2+^ entry. *In situ* hybridization (ISH) and immunohistochemistry (IHC) were used to determine the localization of CaSR mRNA and protein in hair cells. Morpholino oligonucleotides (MOs) were injected to knock down CaSR protein expression. The results clearly indicate that the addition of external Ca^2+^ and a CaSR activator (R-568) suppressed Ca^2+^ influx at the hair bundles. Furthermore, we found that treatment with R-568 attenuated neomycin-induced hair cell death.

## Materials and Methods

### Zebrafish

Adult zebrafish (*Danio rerio*, AB strain) were reared in circulating dechlorinated tap water at 28°C, with a 14-h light/10-h dark photoperiod. Fertilized eggs were incubated in artificial normal water (NW) containing 0.5 mM NaCl, 0.2 mM CaSO_4_, 0.2 mM MgSO_4_, 0.16 mM KH_2_PO_4_, and 0.16 mM K_2_HPO_4_ (pH 7.0). The solutions were prepared by adding various salts (Sigma-Aldrich, St. Louis, MO, United States) to double-distilled water. Larvae were not fed, and the NW was changed daily to ensure optimal water quality during the experiments. The experimental protocols were approved by the Taipei Medical University Animal Care and Utilization Committee (Approval No. LAC-2015-0368).

### Whole-Mount ISH

For ISH, primers (forward: 5′-AAGACGGGCGATATCCTGCTTGGA-3′; reverse: 5′-TGCTCGATGATGGCAGCCATGGC-3′) were used in polymerase chain reaction (PCR) to obtain DNA fragments of zebrafish *casr* (nucleotides 482–1008; XM_684005). The fragments were then inserted into a pGEM-T Easy vector (Promega, Madison, WI, United States). The inserted fragments were amplified through PCR using T7 and SP6 primers, and the products were used as templates for *in vitro* transcription by using T7 or SP6 RNA polymerase (Roche, Mannheim, Germany) in the presence of digoxigenin (DIG)-UTP (Roche) to synthesize sense (T7) and antisense (SP6) probes. The sizes of 3λ DIG-labeled RNA probes were examined using 1% agarose gels, and probe quality and concentrations were determined using dot blot assays. For the dot blot assays, synthesized probes and standard RNA probes were spotted on a nitrocellulose membrane according to the manufacturer’s instructions. After cross-linking and blocking, the membrane was incubated with an alkaline phosphatase-conjugated anti-DIG antibody and stained with nitro blue tetrazolium (NBT) and 5-bromo-4-chloro-3-indolyl phosphate (BCIP) (Roche, Mannheim, Germany).

Zebrafish larvae were anesthetized on ice and fixed with 4% paraformaldehyde in phosphate-buffered saline (PBS; 1.4 mM NaCl, 0.2 mM KCl, 0.1 mM Na_2_HPO_4_, and 0.002 mM KH_2_PO_4_; pH 7.4) at 4°C overnight. Samples were subsequently washed with diethylpyrocarbonate-treated PBST (PBS with 0.1% Tween-20) several times (10 min/wash). After washing, samples were first incubated with a hybridization buffer [HyB, 50% formamide, 5× saline-sodium citrate (SSC), and 0.1% Tween 20] at 65°C for 5 min and then with HyB containing 500 μg/mL yeast transfer tRNA at 65°C for 4 h before hybridization. After hybridization with 100 ng/mL DIG-labeled antisense or sense RNA probes overnight, larvae were serially washed with 50% formamide–2× SSC (at 65°C for 20 min), 2× SSC (at 65°C for 10 min), 2× SSC (at 65°C for 10 min), 0.2× SSC (at 65°C for 30 min, twice), and PBST (at room temperature for 10 min). Larvae were then immunoreacted with an alkaline phosphatase-coupled anti-DIG antibody (1:8000) and stained with NBT and BCIP.

### Whole-Mount Immunocytochemistry

For immunocytochemical staining of the CaSR, zebrafish larvae were first fixed with 4% paraformaldehyde in PBS for 2 h at 4°C. After fixation, the larvae were briefly rinsed with PBS and were then gradually dehydrated with 100% methanol. Following rehydration with PBS, the larvae were blocked with 3% bovine serum albumin for 1 h. The larvae were then incubated with an rabbit anti-CaSR polyclonal antibody (1:500; Herberger and Loretz, 2013; Kwong et al., 2014) or a rabbit anti-S100 polyclonal antibody (1:250; Dako, Carpinteria, CA, United States) in PBS at 4°C overnight. Subsequently, the larvae were washed with PBS for 20 min and incubated with an Alexa Fluor 488 goat anti-rabbit antibody (1:200; Invitrogen) for 2 h in the dark at room temperature. For double immunocytochemical staining, the larvae were incubated overnight at 4°C with CaSR polyclonal and mouse anti-actin monoclonal antibodies (1:100; Chemicon, Temecula, CA, United States). After washing, samples were incubated with Alexa Fluor 568 goat anti-mouse and Alexa Fluor 488 goat anti-rabbit antibodies for 2 h at room temperature. Images were acquired using an upright microscope (Imager M1, Carl Zeiss, Oberkochen, Germany) or a Leica TCS-SP5 confocal laser scanning microscope (Leica Lasertechnik, Heidelberg, Germany).

### Microinjection of Antisense MOs

Morpholino-modified antisense oligonucleotides were purchased from Gene Tools (Philomath, OR, United States). Zebrafish *casr* MO1 (5′-AGTTGGAACCTAATGTGGTCTTCAT-3′; nt -31 to -7; Lin et al., 2014) and *casr* MO2 (5′-ACT TCA GAT GAA ACC TCA TTGCTT C-3′; nt -6 to 19; Kwong et al., 2014) were prepared with sterile water. A standard control MO was provided by Gene Tools; it had no target and no significant biological activity. Following the procedures described in previous studies (Kwong et al., 2014; Lin et al., 2014), the MO solution (4 ng/embryo) containing 0.1% phenol red (as a visual indicator) was injected into zebrafish embryos at the one-to-two-cell stage by using an IM-300 microinjection system (Narishige Scientific Instrument Laboratory, Tokyo, Japan).

### Scanning Ion-Selective Electrode Technique (SIET)

The SIET (Faszewski and Kunkel, 2001; Garber et al., 2005) was used to measure Ca^2+^ influx at the apices of neuromasts. Glass capillary tubes (no. TW 150-4 with 1.12- and 1.5-mm inner and outer diameters, respectively; World Precision Instruments, Sarasota, FL, United States) were pulled on a Sutter P-97 Flaming Brown pipette puller (Sutter Instruments, San Rafael, CA, United States) into micropipettes with tip diameters of 3–4 μm. The micropipettes were then baked at 120°C overnight and were coated with dimethyl chlorosilane (Sigma-Aldrich) for 30 min. A Ca^2+^-selective microelectrode was fabricated as previously described (Garber et al., 2005; Lin et al., 2013). To create a Ca^2+^-selective microelectrode, the micropipettes were backfilled with a 1-cm column of electrolyte (100 mM CaCl_2_) and frontloaded with a 20–30-μm column of Ca^2+^ ionophore I cocktail A (Sigma-Aldrich).

Details of the system are provided in our previous studies (Wu et al., 2010; Shih et al., 2012; Lin et al., 2013, 2015; Horng et al., 2015). The ion-selective microelectrode was connected to the main amplifier with a Ag/AgCl wire electrode holder and preamplifier (Applicable Electronics, East Falmouth, MA, United States), and the circuit was completed with a salt bridge (3 M KCl in 3% agarose connected to a Ag/AgCl wire). A step-wise motor-driven three-dimensional (3D) positioner (Applicable Electronics) was attached to an upright microscope (BX-50WI, Olympus, Tokyo, Japan) and was used to oscillate and position the microelectrode. A 10× dry and 60× water-immersion objective lens (working distance: 3.3 mm) were used. The microscope was equipped with a digital camera (ILCE-6300, Sony, Japan) that enabled imaging and recording on a monitor. Automated Scanning Electrode Technique (ASET) software (Science Wares, East Falmouth, MA, United States) was used for data acquisition, preliminary processing, and controlling the 3D electrode positioner. The microelectrode was oscillated with an excursion distance of 10 μm, and a typical cycle was completed in 3–4 s. The voltage measurement recorded nearest to the tissue was subtracted from the measurement recorded at the opposite end of the cycle. This subtraction served as a self-referencing feature of the probe.

### Calibration of the Ion-Selective Microelectrode

Before collecting biological data, the efficiency of the Ca^2+^ probe was determined using a method described in a previous study (Garber et al., 2005). The Nernstian properties of each electrode were measured by positioning the Ca^2+^ microelectrode in a series of standard solutions (0.1, 1, 10, and 100 mM CaCl_2_). Linear regression yielded a Nernstian slope of 30 ± 0.53 (*n* = 10) for Ca^2+^, which was obtained by plotting the voltage output of the probe against log[Ca^2+^] values. According to technical documents provided by Sigma, the selectivity coefficients of the Fluka Ca^2+^ ionophore I cocktail A are approximately 1000-times more selective for Ca^2+^ than for Mg^2+^.

### Measurement of Ca^2+^ Flux at Neuromasts

The SIET was performed at room temperature (26–28°C) in a small plastic recording chamber filled with 1 mL of recording medium that contained NW, 300 μM MOPS buffer, and 0.1 mg/L ethyl 3-aminobenzoate methanesulfonate (tricaine, Sigma-Aldrich). The pH of the recording medium was adjusted to 7.0 by NaOH or HCl addition. Before each measurement, an anesthetized 4-day postfertilization (dpf) larva was placed in a 1 mm-wide trench of the chamber and observed through a 60× water-immersion lens. The microelectrode was then placed in the recording medium and was positioned at the apical surface of the larva’s L1 neuromast, where stereocilia are located, to record Ca^2+^ activity (Ghysen and Dambly-Chaudiere, 2007; Haehnel et al., 2012). The microelectrode was oscillated orthogonally for 10 μm to deflect the kinocilia and to record Ca^2+^ influx (Lin et al., 2013). The microelectrode was tested in several positions to identify the strongest signal. Ca^2+^ influxes at neuromasts were measured for five to ten replicates, and the median value was employed to calculate ionic fluxes by using ASET software (Applicable Electronics). Briefly, voltage gradients measured using ASET software were converted into concentration (activity) gradients by using the following equation: Δ*C* = *C*_b_ × 10^(ΔV/S)^ -*C*_b_. ΔC (measured in μmole⋅L^-1^⋅cm^-3^) represents the concentration gradient between the two points. *C*_b_ (measured in μmole⋅L^-1^) represents the background ion concentration, which is calculated as the average of the concentration at each point. ΔV (in μV) denotes the voltage gradient obtained from ASET. S indicates the Nernst slope of the electrode. The concentration gradient was subsequently converted into (extracellular) ion flux according to Fick’s law of diffusion and using the following equation: *J* = *D*(ΔC)/Δ*X*. *J* (in pmol⋅cm^-2^⋅s^-1^) denotes the net flux of the ion. *D* is the diffusion coefficient, which is 8 × 10^-6^ cm^2^⋅s^-1^ for Ca^2+^. Δ*C* (in pmol⋅cm^-3^) represents the concentration gradient. Δ*X* (in cm) indicates the distance between the two points. The SIET was performed on the L1 neuromasts (Ghysen and Dambly-Chaudiere, 2007; Haehnel et al., 2012) of the posterior lateral lines in zebrafish larvae. Before SIET measurements, larvae were preincubated in drug medium for 30 min. Thereafter, larvae were placed in the drug-free recording medium and Ca^2+^ influxes of L1 neuromasts were measured. One L1 neuromast per larva was examined.

### Scanning Electron Microscopy

Embryos were fixed overnight at 4°C in PBS-buffered 4% paraformaldehyde–5% glutaraldehyde. After being rinsed with PBS, specimens were postfixed with 2% osmium tetraoxide in 0.1 M cacodylate buffer for another 2 h. After being rinsed with cacodylate buffer and dehydrated with ethanol, specimens were critical point dried with liquid CO_2_ in a K850 critical point drier (Quorum Technologies Ltd., East Grinstead, United Kingdom) and were sputter coated for 3 min with a gold-palladium complex in a vacuum evaporator (IB-2; Hitachi, Ltd., Tokyo, Japan). Coated specimens were examined using scanning electron microscopy (SEM; Hitachi S-2400, Tokyo, Japan). The hair bundle of L1 neuromasts in the posterior lateral lines were observed.

### Drug Preparation and Treatment

Neomycin (10 mg/mL, Sigma-Aldrich) was dissolved in NW to a final concentration of 1 or 10 μM (pH 7.0). R-568 (Santa Cruz Biotechnology) was dissolved in DMSO to a stock concentration of 10 mM, and adequate stock was dissolved in NW to final concentrations of 2–16 μM. The final concentration of DMSO in the working solutions was <0.1%. In R-568 experiments, the 0 R-568 treatment group was incubated in medium containing 0.1% DMSO. Larvae were immersed in the drug medium for 30 min. Thereafter, larvae were immersed in the drug-free recording medium and were measured using the SIET. CaSO_4_ was dissolved in NW to a final concentration of 2 mM to prepare HCa NW. The pH of media was adjusted to 7.0. For hair-cell-counting experiments, larvae were incubated in neomycin for 1 h. For R-568 and neomycin incubation, larvae were preincubated in R-568 for 10 min, followed by incubation in R-568 and neomycin. After treatments, larvae were washed with NW, and hair cells were labeled with rhodamine 123 (Sigma) for 10–15 min or FM1-43 (Molecular Probes) for 2 min. Rhodamine 123 was dissolved in ethanol to a stock concentration of 2.6 mM and then dissolved in NW to a final concentration of 2.6 μM. Similarly, FM1-43 was dissolved in DMSO to a stock concentration of 3 mM and then dissolved in NW to a final concentration of 3 μM. Live larvae were observed under an upright microscope (BX60; Olympus) equipped with a digital camera (Canon 50D). The hair cell numbers of L1 neuromasts in the posterior lateral lines were determined (Ghysen and Dambly-Chaudiere, 2007; Haehnel et al., 2012). One L1 neuromast per larva was examined.

### Statistical Analysis

Data are presented as mean ± standard error (SE). Values obtained under each condition were analyzed using one-way analysis of variance (ANOVA), followed by Tukey’s pairwise comparisons. Student’s unpaired *t*-test (two-tailed) was used for simple comparisons of two means. In all cases, significance was accepted at a level of 0.05. All experiments were repeated for at least three independent trials to confirm results, and representative data from one of the trial are displayed in the figures.

## Results

### Localization of the CaSR in Neuromast Hair Cells of Zebrafish Larvae

**FIGURE 1:**
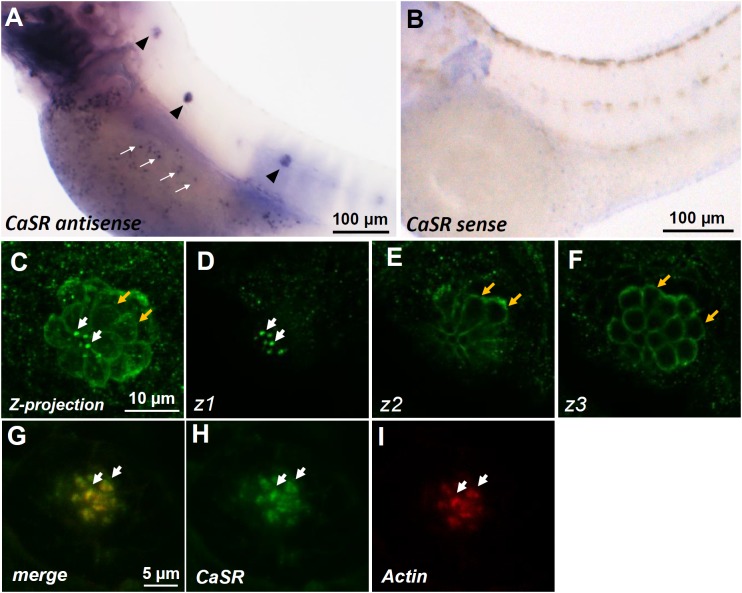
*In situ* hybridization and immunocytochemistry of the Ca^2+^-sensing receptor (CaSR) in zebrafish larvae. CaSR mRNA was expressed in neuromasts (arrowheads) and ionocytes (arrows) of 3- day-postfertilization (dpf) larvae **(A)**. The sense probe detected no signal in larval skin **(B)**. Confocal images of CaSR antibody-labeled lateral-line neuromasts of 4-dpf larvae (**C**, a Z-projection). Confocal *z*-stacks of the same neuromast revealed that the CaSR was expressed in stereocilia (white arrows in **C,D**) and basolateral membranes (orange arrows in **C,E,F**). Double immunocytochemical labeling of the CaSR **(H)** and actin **(I)** in 4-dpf larvae; the merged image is shown in **(G)**. White arrows indicate the colocalization of both CaSR and actin signals in stereocilia.

Whole-mount ISH was used to determine the localization of *casr* mRNA in 3-dpf zebrafish larvae. Signals of *casr* mRNA were identified in the lateral-line neuromasts of larvae (arrowheads in **Figure 1A**) and in ionocytes dispersed on the yolk sac skin (white arrows in **Figure 1A**). These signals were not detected in the negative control reacted with the sense probe (**Figure 1B**). Whole-mount IHC was further used to determine the localization of the CaSR protein in 4-dpf larval neuromasts. Confocal microscopic images revealed CaSR signals in the stereocilia (white arrows in **Figures 1C,D**) and basolateral membranes of hair cells (yellow arrows in **Figures 1C,E,F**). Optical sections in various focal planes (z1: at stereocilia; z2: at 3 μm below the stereocilia; z3: at 6 μm below the stereocilia) are, respectively, presented in **Figures 1D–F**. Double IHC was used to identify coexpressed CaSR and actin in hair cell stereocilia (**Figures 1G–I**).

### Effects of the CaSR Activator (R-568) on Ca^2+^ Influx at Neuromasts

**FIGURE 2:**
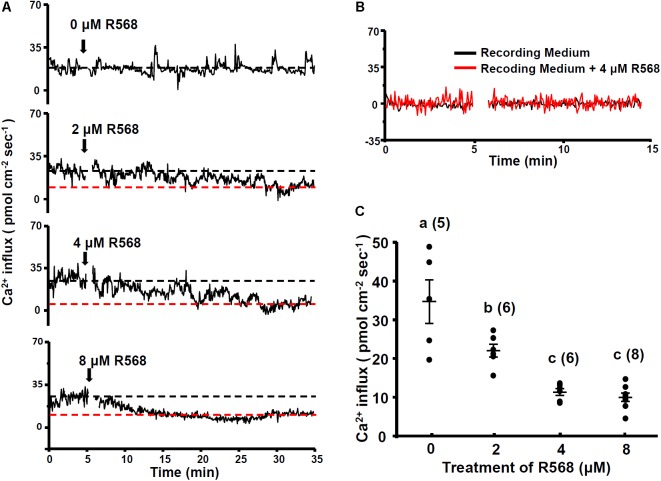
Effect of R-568 on neuromast Ca^2+^ influxes. Sequential recordings of L1 neuromast Ca^2+^ influxes of 4-dpf larvae before and after the addition of 0, 2, 4, and 8 μM R-568 (arrows in **A**). The black dotted line denotes the average Ca^2+^ influx (time: 3–5 min) before the addition of the drug. The red dotted line denotes the average Ca^2+^ influx 30 min after the addition of the drug (time: 30–33 min). **(B)** Illustrates the fluxes of the background signal in recording medium (black line) and the recording medium with R-568 (red line; no fish). **(C)** Larvae were preincubated in normal water with 2, 4, or 8 μM R-568 for 30 min. The larvae were then placed in the normal recording medium without R-568, and Ca^2+^ influxes at L1 neuromasts were recorded. Data are presented as mean ± SE. ^a,b,c^ indicate significant difference (by one-way ANOVA and Tukey’s comparison, *p* < 0.05). Numbers in parentheses are the numbers of neuromasts. One L1 neuromast per larva was examined.

To investigate the role of the CaSR in Ca^2+^ influx at the apical surface of hair cells, 4-dpf larvae were treated with a CaSR activator (R-568). R-568 has been shown to activate the CaSR in mammalian and fish studies (Greenwood et al., 2009; Lu et al., 2009). Real-time recordings of Ca^2+^ influxes at neuromast hair cells of larvae treated with 0, 2, 4, and 8 μM R-568 are presented in **Figure 2A**. Ca^2+^ influx was detected before the addition of R-568, but the influx gradually declined after the addition of R-568 (arrows in **Figure 2A**). Treatment with 2, 4, and 8 μM R-568, respectively, reduced Ca^2+^ influxes by 37, 66, and 74% (**Figure 2C**). **Figure 2B** presents background fluxes (without larva).

### Effect of CaSR Knockdown on Ca^2+^ Influx at Neuromasts

**FIGURE 3:**
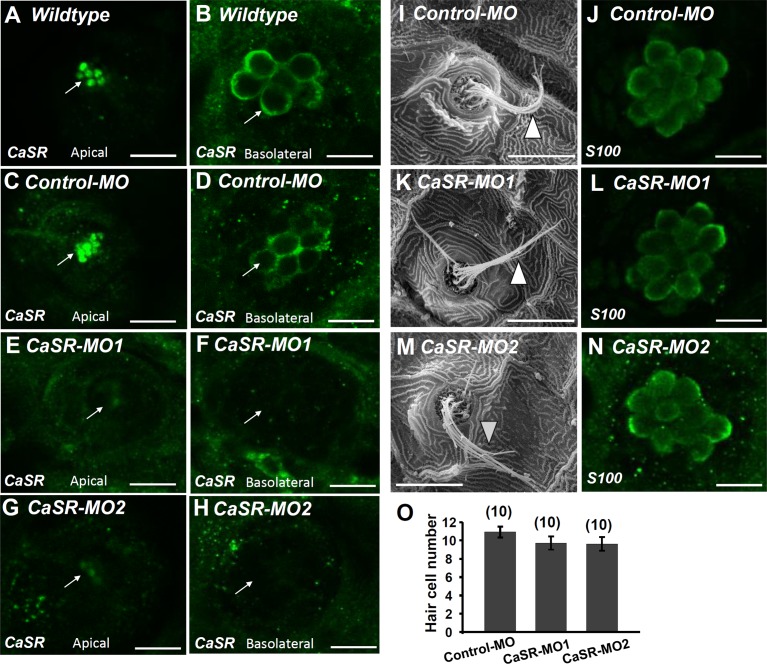
Ca^2+^-sensing receptor (CaSR) protein expression in neuromast hair cell of CaSR morpholino oligonucleotide (MO) knockdown larvae. Immunocytochemical staining with CaSR antibodies was conducted in 4-dpf wild-type larvae **(A,B)**, control MO-injected larvae (control-MO; **C,D**), and CaSR-MO-injected larvae (CaSR-MO; **E–H**). CaSR signals (green) were detected in the apical (arrows; **A,C**) and basolateral (arrows; **B,D**) membranes of hair cells in wild-type and control-MO larvae. In CaSR-MOs, the CaSR signal was weak in the apical and basolateral membranes **(E–H)**. Scanning electron microscopy images revealed the morphologies of L1 neuromat hair bundles in 4-dpf control-MOs and CaSR-MOs (arrowheads; **I,K,M**). Immunocytochemical staining with S100 antibodies was conducted in 4-dpf control-MOs and CaSR-MOs **(J,L,N)**. The hair cell numbers of L1 neuromasts in the posterior lateral lines were determined **(O)**. Data are presented as mean ± SE. No significant differences were identified (one-way ANOVA and Tukey’s comparison, *p* < 0.05). Numbers in parentheses are the numbers of neuromasts. One L1 neuromast per larva was examined.

CaSR-specific MOs were used to knock down the protein expression of the CaSR. No obvious difference was observed in the phenotype of CaSR MO-injected zebrafish embryos (CaSR morphants) and control MO-injected embryos (control morphants) or wild-type embryos. CaSR protein expression in 4-dpf morphants was analyzed using IHC. CaSR signals were found in the stereocilia and basolateral membranes of hair cells in wild-type embryos (**Figures 3A,B**) and control morphants (**Figures 3C,D**). The signals were remarkably lower in CaSR morphants (**Figures 3E–H**). SEM was used to examine the L1 hair bundle morphology of neuromasts. No obvious difference was identified in neuromast kinocilia and apical membrane appearance between 4-dpf CaSR morphants and control morphants (**Figures 3I,K,M**). The S100 antibody was used to label neuromast hair cells in zebrafish, and no significant difference was found in the morphology and number of hair cells in the L1 neuromast between 4-dpf control morphants and CaSR morphants (**Figures 3J,L,N,O**).

**FIGURE 4:**
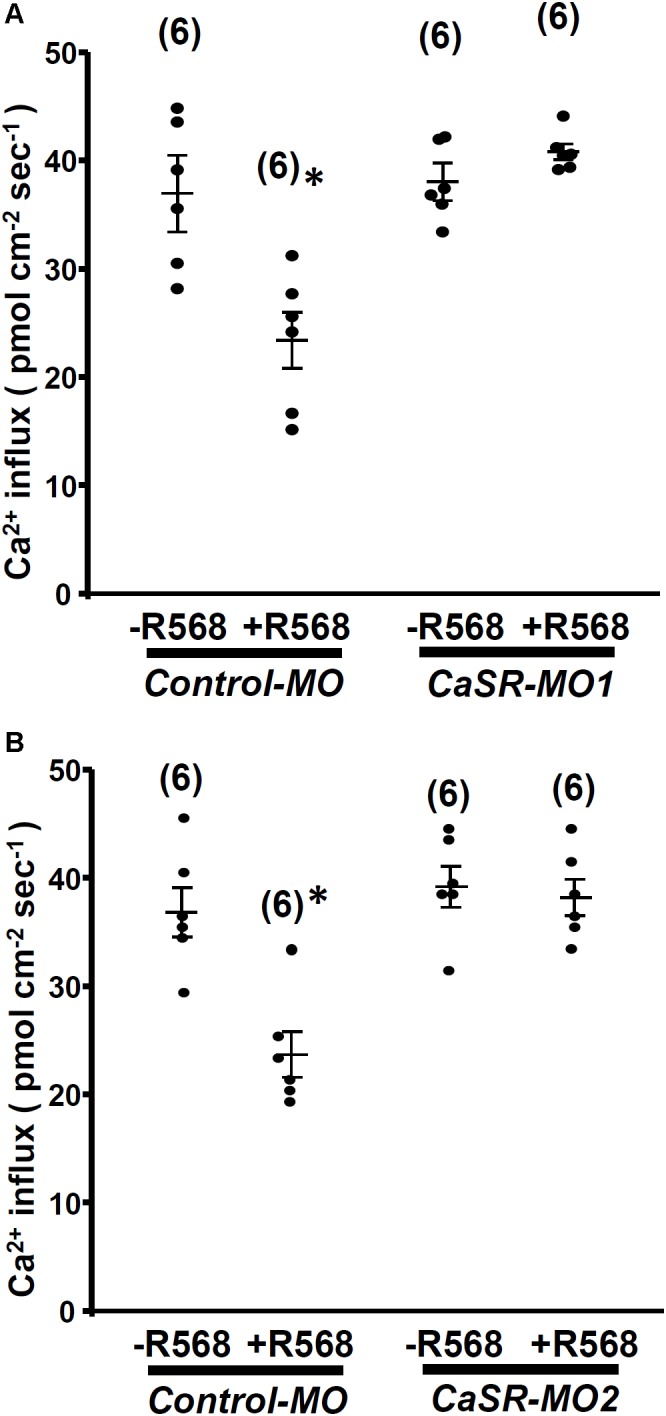
Effect of R-568 on Ca^2+^ influx at neuromasts in Ca^2+^-sensing receptor (CaSR) morpholino-oligonucleotide (MO)-injected larvae. In 4-dpf control MO-injected larvae (control-MO), treatment with 2 μM R-568 reduced Ca^2+^ influxes at L1 neuromast hair cells. However, treatment with 2 μM R-568 did not alter neuromast hair cell Ca^2+^ influxes in CaSR-MO1- **(A)** or MO2-**(B)** injected larvae. Data are presented as mean ± SE. ^∗^Significant difference from the -R-568 group (without R-568; Student’s *t*-test, *p* < 0.05). Numbers in parentheses are the numbers of neuromasts. One L1 neuromast per larva was examined.

No significant difference was observed in L1 neuromast Ca^2+^ influxes between 4-dpf CaSR morphants and control morphants. Furthermore, R-568 (2 μM) treatment reduced Ca^2+^ influxes in control morphants, but not in CaSR morphants (**Figure 4**). The results indicate that CaSR knockdown through MOs did not affect Ca^2+^ influxes in hair cells but neutralized the effect of R-568.

### Effect of HCa Exposure on Ca^2+^ Influx at Neuromasts

**FIGURE 5:**
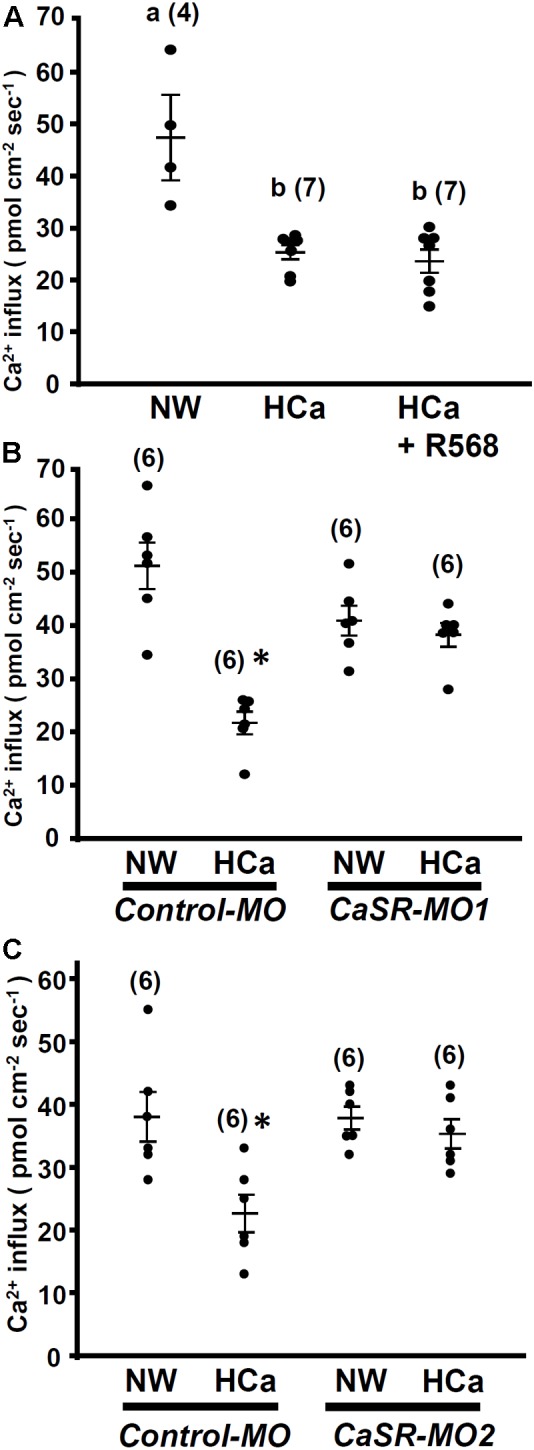
High external Ca^2+^ alters neuromast Ca^2+^ influxes. 4-dpf larvae were preincubated in normal water (NW; 0.2 mM Ca^2+^) or HCa NW (HCa; 2 mM Ca^2+^) for 30 min. After preincubation, larvae were placed in normal recording medium, and L1 neuromast Ca^2+^ influxes were recorded. After HCa treatment, neuromast hair cell Ca^2+^ influxes decreased **(A)**. Incubation in HCa medium containing 2 μM R-568 did not suppress Ca^2+^ influx more than HCa treatment alone did **(A)**. In control morpholino oligonucleotide (MO)-injected larvae (control-MO; **B,C**), HCa preincubation decreased Ca^2+^ influx at neuromast hair cells. In Ca^2+^-sensing receptor (CaSR) MO1- **(B)** and MO2- **(C)** injected larvae, HCa exerted no effect on Ca^2+^ influxes at neuromast hair cells. Data are presented as mean ± SE. A significant difference was found between ^a^ and ^b^ (by one-way ANOVA and Tukey’s comparison, *p* < 0.05). ^∗^Significant difference from the NW group (Student’s *t*-test, *p* < 0.05). Numbers in parentheses are the numbers of neuromasts. One L1 neuromast per larva was examined.

In this experiment, 4-dpf larvae were incubated in HCa water (2 mM Ca^2+^) for 30 min and were then transferred to the recording medium to measure Ca^2+^ influxes at L1 neuromasts. Similar to R-568 treatment (**Figure 2**), HCa treatment suppressed Ca^2+^ influxes by 47%. Treatment with a combination of HCa and R-568 (2 μM) did not suppress Ca^2+^ influxes more than did HCa treatment alone (**Figure 5A**).

HCa treatment also suppressed Ca^2+^ influxes in control morphants, but not in CaSR morphants (**Figures 5B,C**).

### R-568 Attenuated Neomycin-Induced Hair Cell Death

**FIGURE 6:**
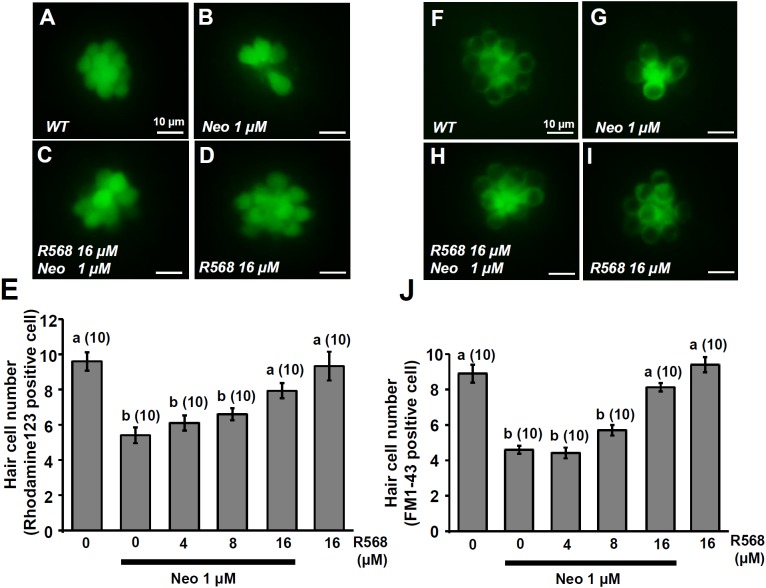
Effect of R-568 on 1 μM neomycin-induced hair cell death. 4-dpf larvae were incubated in 1 μM neomycin media with various levels of R-568 **(E,J)** for 1 h. After treatment, neuromast hair cells were labeled with rhodamine 123 **(A–E)** or FM1-43 **(F–J)**. R-568 at 16 μM neutralized the neuromast hair cell death caused by 1 μM neomycin **(E,J)**. Data are presented as mean ± SE. A significant difference was found between ^a^ and ^b^ (by one-way ANOVA and Tukey’s comparison, *p* < 0.05). Numbers in parentheses are the numbers of neuromasts. One L1 neuromast per larva was examined.

**FIGURE 7:**
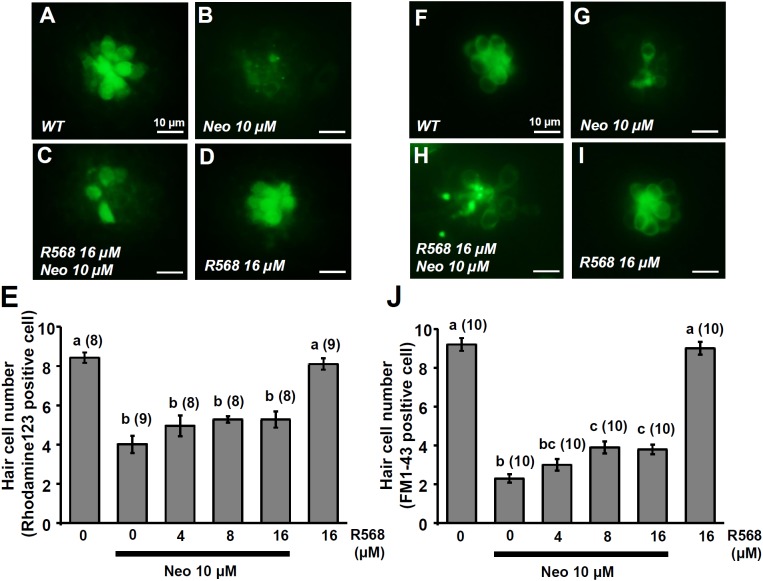
Effect of R-568 on 10 μM neomycin-induced hair cell death. 4-dpf larvae were incubated in 10 μM neomycin media with various levels of R-568 **(E,J)** for 1 h. After incubation, neuromast hair cells were labeled with rhodamine 123 **(A–E)** or FM1-43 **(F–J)**. The addition of 16 μM R-568 partial neutralize the neuromast hair cell death induced by 10 μM neomycin (J). Data are presented as mean ± SE. A significant difference was found between values with superscript letters (^a,b,c^; by one-way ANOVA and Tukey’s comparison, *p* < 0.05). Numbers in parentheses are the numbers of neuromasts. One L1 neuromast per larva was examined.

In this experiment, two fluorescent dyes, FM1-43 and rhodamine 123 (Rho-123), were used to label living hair cells in 4-dpf zebrafish larvae (Johnson et al., 1980; Hu, 2007). Exposure of larvae to 1 μM neomycin for 1 h decreased the numbers of Rho-123- and FM1-43-labeled L1 neuromast hair cells by 47% and 50%, respectively (**Figure 6**). The treatment of 1 μM neomycin-exposed larva with R-568 at a concentration of 16 μM, but not at lower doses (2, 4, and 8 μM), was found to effectively neutralize the effects of neomycin on hair cells (**Figures 6E,J**). Treatment with 16 μM R-568 alone did not affect the number of hair cells. Treatment with R-568 at a concentration of 16 μM was found to partial neutralize the effects of 10 μM neomycin (**Figure 7J**).

## Discussion

The SIET was developed to enable the non-invasive measurement of ion flux in tissues or cells (Garber et al., 2005). Jaffe and Nuccitelli (1974) first developed and applied the “vibrating probe” or “vibrating voltage probe” technique to biological systems, and the technique was later modified into the SIET (Adlimoghaddam et al., 2014; D’Silva et al., 2017), non-invasive microelectrode ion flux estimation technique (Shabala et al., 2006; Koseki et al., 2012), and the non-invasive micro-test technique. To date, these techniques have been applied to various systems, including plants (Ma et al., 2015; Lei et al., 2017), *Caenorhabditis elegans* (Adlimoghaddam et al., 2014), glioma cells (Hu et al., 2014), *Escherichia coli* (Shabala et al., 2006; Koseki et al., 2012), and mosquito larvae (Boudko et al., 2001; D’Silva et al., 2017). In previous studies, using the non-invasive SIET, we analyzed ion fluxes in intact zebrafish and medaka larvae (Wu et al., 2010; Shih et al., 2012; Lin et al., 2013, 2015; Horng et al., 2015). We also demonstrated that the SIET is suitable for studying hair cells in intact zebrafish larvae (Lin et al., 2013, 2015). The SIET can be used to detect specific ion fluxes at the surface of neuromasts, where hair bundles are located. The “vibrating” feature of the SIET may be used to simultaneously stimulate a hair bundle mechanically and record ion fluxes. Alternative electrophysiological approaches can be used to determine MET channel activity. Measurements of microphonic potential represent the overall voltage response of hair cells but don’t reveal the specific types of ion activity involved in mechanotransduction (Olt et al., 2016). Researchers have applied the single-cell patch-clamp technique to measure the basolateral electrical activities of hair cells after removing supporting and mantle cells (Ricci et al., 2013; Olt et al., 2016).

The MO is used for gene knockdown in a range of model animals, including *Xenopus*, zebrafish, sea urchin, and chick. Guidelines for MO use in zebrafish have been published to help researchers distinguish specific phenotypes from off-target effects (Stainier et al., 2017). According to suggestions in the guidelines, we used three MOs (control-MO, UTR MO [MO1], and ATG MO [MO2]) to evaluate the effect of *casr* knockdown in zebrafish embryos. Control MO-injected embryos did not exhibit developmental abnormalities. The doses of MO1 and MO2 used in this study did not result in significant mortality or abnormal behavior in the morphants, and this result is consistent with those of previous studies (Kwong et al., 2014; Lin et al., 2014). The neuromast hair cell morphology of MO1- and MO2-injected morphants was not significantly different from that of control morphants and wild-type embryos (**Figure 3**). CaSR protein expression in *casr* morphants was lower than in control and wild-type embryos, and Ca^2+^ influxes at neuromast hair cells was affected by the MOs (**Figures 3**–**5**). These results demonstrated that the MOs used in this study resulted in minimal off-target effects, and hair cell function was impaired after CaSR knockdown.

The MET channels expressed at the tips of stereocilia are cation-permeable and exhibit high selectivity for Ca^2+^. Research for MET channel identification has been conducted for more than 20 years, and several candidates have been proposed, including analogs of the epithelial Na^+^ channel, multiple transient receptor potential channels (TRPN, TRPC, and TRPML; Fettiplace and Kim, 2014), and transmembrane channel-like proteins (Fettiplace, 2016). Deflection of a hair bundle tenses the tip link and activates the MET channel. Open MET channels reclose via an initial fast adaptation mechanism followed by a much slower, myosin-based motor process, both of which are driven by Ca^2+^ entry through the channel itself (Holt et al., 2002; Stauffer et al., 2005; Fettiplace and Kim, 2014). A previous study also demonstrated that Ca^2+^ acts as a permeable blocker of the channel, decreasing total MET current (Ricci and Fettiplace, 1998). Ca^2+^ is then exported back to the endolymph by PMCA, which is present in high concentrations in the stereociliary membrane (Fettiplace and Kim, 2014). In a previous study, the alteration of endolymph Ca^2+^ homeostasis and the absence of PMCA2 in various mutants caused the disruption of the MET process and led to deafness (Giacomello et al., 2012).

Zebrafish are exposed to various Ca^2+^ concentrations in their natural environment, from 0.01 mM (soft water) to 3 mM (hard water) (Hoenderop et al., 2005; Pan et al., 2005; Lin and Hwang, 2016; USGS Office of Water Quality, 2017). Studies of fish have indicated strong CaSR expression in gills and the olfactory epithelium (Loretz et al., 2004; Loretz, 2008). CaSR expression at the body surfaces of fish may enable the direct measurement of environmental Ca^2+^ concentrations. Ionocytes in fish gills and embryos proliferate in response to reduced environmental Ca^2+^ concentrations (Uchida et al., 2002; Pan et al., 2005). Extracellular recording from the olfactory bulb of goldfish through electroencephalogram showed that the olfactory system is acutely sensitive to changes in external freshwater Ca^2+^ concentrations (0.05–3 mM; Hubbard et al., 2002). These findings suggest that the CaSR may be involved in the functional regulation of gills and olfactory systems during physiological responses to changes in Ca^2+^ concentrations. Studies of hair cells have found that extracellular Ca^2+^ is critical for MET channel function (Richardson and Russell, 1991; Coffin et al., 2009; Lin et al., 2013). In the present study, we demonstrated CaSR protein expression in hair bundles and suggested that the CaSR senses high environmental Ca^2+^ and reduces Ca^2+^ influx at stereocilia.

The CaSR activation increases PMCA activity in mouse mammary epithelial cells (VanHouten et al., 2007). HEK-293 cells expressing activating CaSR variants show reduced PMCA expression (Ranieri et al., 2013). Hence, PMCA is a potential downstream target of the CaSR. PMCA expression has been observed in rat hair cell stereocilia and basolateral membranes (Chen et al., 2012). In zebrafish, the PMCA mRNA expression pattern recapitulates the GFP expression in the Tg:*atp2b1a*-GFP line (Go et al., 2010). However, no clear evidence has demonstrated that PMCA protein expression occurs in stereocilia. Thus, the speculation that PMCA protein expression occurs in zebrafish stereocilia and is regulated by the CaSR requires further investigation. If PMCA is expressed in the stereocilia of zebrafish hair cells, we cannot exclude the possibility that CaSR activation increases PMCA activity. Thus, the effects we observed in this study may be a combined result of reduced MET-channel-mediated Ca^2+^ influx and increased PMCA-mediated Ca^2+^ efflux. Therefore, the functional roles of the CaSR, PMCA, and MET channels in zebrafish hair cells remain to be determined.

To investigate the function of the CaSR, zebrafish larvae were treated with R-568 or HCa, both of which suppress MET-channel-mediated Ca^2+^ influx in hair cells (**Figures 2**, **5**). Treatment with a combination of R-568 and HCa^2+^ did not enhance the inhibition of Ca^2+^ influx, suggesting that the CaSR may mediate the effect of HCa^2+^ on hair cells. In the CaSR morpholino knockdown experiments, the inhibitory effect of R-568 on the MET channel was neutralized (**Figure 4**). The results suggest that R-568 acts through the CaSR rather than through the MET channel, excluding the possibility that R-568 might directly block the MET channel.

Previous studies have indicated that neomycin is taken up by hair cells through the MET channel, leading to cell death (Marcotti et al., 2005; Alharazneh et al., 2011). Blockers of MET channels (curare, quinine, and amiloride) significantly reduce gentamicin uptake and prevent hair cell death (Alharazneh et al., 2011). Low extracellular Ca^2+^ and noise exposure increase the opening probability of MET channels and neomycin uptake, which induce hair cell death (Farris et al., 2006; Coffin et al., 2009; Li and Steyger, 2009). High levels of extracellular Ca^2+^ or Mg^2+^ may decrease the opening probability of MET channels and neomycin uptake, which, in turn, may protect hair cells from neomycin-induced death (Richardson and Russell, 1991; Coffin et al., 2009). In the present study, treatment with 2 μM of a CaSR activator (R-568) significantly suppressed MET-channel-mediated Ca^2+^ influx (**Figure 2**), suggesting that R-568 played a protective role during neomycin treatment. Our data indicate that 16 μM R-568 completely and partially neutralized hair cell death-inducing effects caused by 1 and 10 μM neomycin, respectively (**Figures 6**, **7**). Previous studies have found that HCa^2+^ (2 mM) can neutralize damage to hair cells caused by 100 μM neomycin (Coffin et al., 2009; Lin et al., 2013). Our results suggest that CaSR stimulation contributes to a part of the protective effect of HCa^2+^.

The fluorescent dyes DASPEI (which is used to label mitochondria), YO-PRO1 (which is used to label DNA), and FM1-43 are frequently used to label hair cells (Ou et al., 2010). In the present study, we stained larvae with rhodamine 123, a mitochondrial dye, for the first time. Rhodamine-123-labeled hair cells were distinguishable and countable, and the data were comparable with those for FM1-43 staining. Hence, in this study, we demonstrated that rhodamine 123 is suitable for labeling hair cells.

Previous studies have indicated that activation of the CaSR opens the Ca^2+^-activated K^+^ channel and non-selective cation channel in endothelial cells, neurons, and astrocytoma cells (Ye et al., 1996; Chattopadhyay et al., 1999a,b; Weston et al., 2005). Researchers have suggested that the CaSR regulates K^+^ channels and TRPC channels through mitogen-activated protein kinase or the phospholipase C (PLC)-PKC pathway (Ye et al., 2004; Chow et al., 2011; Kong et al., 2012). Another study indicated that CaSR stimulation in HEK293 cells activates both PLC and phosphatidylinositol 4-kinase (PI-4-K) and subsequently Kir currents (Liu et al., 2016). In the present study, activation of the CaSR attenuated MET-channel-mediated Ca^2+^ influx in hair cells. In sum, the findings demonstrate that the CaSR senses extracellular Ca^2+^ and thereafter affects the function of the channels through a direct or indirect pathway.

Ca^2+^ is not the only CaSR agonist. Experiments on parathyroid gland and kidney cells have demonstrated that various divalent (e.g., Mg^2+^ and Sr^2+^) and trivalent (La^3+^ and Gd^3+^) cations and aminoglycoside antibiotics (e.g., neomycin) can activate the CaSR (Riccardi and Brown, 2010; Alfadda et al., 2014). Gd^3+^, La^3+^, and aminoglycosides are also used as MET channel blockers (Waguespack and Ricci, 2005; Fettiplace, 2009). The data of the present study do not confirm whether neomycin directly acts on the CaSR in neuromast hair cells. Therefore, the functional mechanism of the CaSR in hair cells requires further investigation.

## Ethics Statement

This study was carried out in accordance with the recommendations of ‘Taipei Medical University Animal Care and Utilization Committee’. The protocol was approved by the ‘Taipei Medical University Animal Care Committee’ (Approval No. LAC-2015-0368).

## Author Contributions

All authors had full access to all the study data and assume responsibility for the integrity of the data and the accuracy of data analysis. L-YL and J-LH: study concept and design, critical revision of the manuscript for intellectual content, funding acquisition, and study supervision. Y-HY and C-HL: acquisition and analysis of data. L-YL, Y-HY, C-HL, and J-LH: interpretation of data. L-YL, G-YH, P-PH, and J-LH: drafted the manuscript. Y-HY, G-YH, and P-PH: administrative, technical, and material support.

## Conflict of Interest Statement

The authors declare that the research was conducted in the absence of any commercial or financial relationships that could be construed as a potential conflict of interest.
